# Association Between Internet Addiction and Dietary Habits Among Omani Junior College Students

**DOI:** 10.18295/squmj.6.2024.032

**Published:** 2024-08-29

**Authors:** Mickael A. Joseph, Jansirani Natarajan, Huda Al-Hinai

**Affiliations:** 1 Fundamentals and Administration, College of Nursing, Sultan Qaboos University, Muscat, Oman; 2 School of Health in Social Science, The University of Edinburgh, Scotland, United Kingdom; 3 Ibri Regional Hospital, Ibri, Oman

**Keywords:** Internet Addiction Disorder, Diet, Students, Appetite, Oman

## Abstract

**Objectives:**

This study aimed to investigate the association between internet addiction and dietary habits among Omani junior college students.

**Methods:**

This cross-sectional study was conducted at Sultan Qaboos University, Muscat, Oman, among junior college students surveyed in November 2020. The Compulsive Internet Use Scale and a dietary habits questionnaire were used. Chi-square cross-tabulation analyses were used to explore the relationship between internet addiction and dietary habits.

**Results:**

A total of 377 students were included in this study. Overall, 59.9% of the junior college students were identified as having an internet addiction. Within this group, 62.8% reported reduced meal sizes and 54.4% reported a decrease in appetite. There was a statistically significant difference in both meal size (X^2^ = 30.528; *P* <0.001) and appetite changes (X^2^ = 28.731; *P* <0.001) among students with different levels of internet addiction.

**Conclusion:**

The results of this study suggest a possible link between internet addiction and altered dietary habits among this population. This study highlights the need for strategies that encourage healthy living behaviours and raise awareness about the adverse effects of internet addiction.

The widespread use of the internet and the growth of digital devices in recent years have significantly changed several aspects of life, including education.^1^ This digital revolution has enabled quick communication across great distances, allowing people to stay in touch with friends, family and co-workers all around the world.^2^ Furthermore, the internet has transformed education by providing an abundance of online resources, e-learning platforms and virtual classrooms. Students can now access a wealth of knowledge and skills from the comfort of their own homes using their mobile devices.^3^ The internet’s huge array of entertainment sources, such as streaming services, social networking and online gaming, have increased leisure activities.^4^ However, the rising prevalence of internet addiction, particularly among younger populations such as junior college students, has emerged as a major global issue.^5^ Internet addiction is defined as compulsive and excessive internet use which has a negative impact on an individual’s physical health, psychological well-being and social interactions.^6^

Internet addiction can result in sedentary behaviour and a decrease in physical activity, which may lead to overweight, obesity and sleep disruptions.^7^–^9^ Internet addiction, particularly in the context of digital gaming, can have a substantial impact on individuals’ eating habits and nutritional practices.^10^ Because of the immersing nature of digital gaming, a phenomenon known as ‘gaming binge’ could occur, in which gamers become profoundly absorbed in extended gaming sessions, often disregarding other activities, including eating.^11^

Several studies have been conducted to investigate the association between internet usage and dietary changes. Hassan and Ahmed studied the dietary habits of Egyptian adolescents suffering from internet addiction and found that compared to their non-addicted peers, these adolescents were more prone to experience loss of appetite and eat large meals at a fast rate.^10^ In a Pakistani study, Waheed *et al*. found internet addiction to be an increasing issue among university students, negatively affecting their dietary patterns.^12^ At a Turkish university, Gündüz *et al*. examined how excessive internet use among university students can negatively affect their daily life and contribute to lifestyle-related issues such as alcohol use.^13^ Furthermore, Stiglic and Viner conducted a systematic review on the association between screen time (including internet usage) and dietary patterns in children and adolescents.^14^ They found an association between higher screentime and increased obesity, unhealthy diet, low quality of life, poor well-being and poor cardiorespiratory fitness. These results show that young people’s less-than-ideal diets may be related to the increased time they spend online, particularly playing video games. Studying the impact of internet addiction on the dietary habits of young university students is essential for identifying its links to health issues, academic performance, mental well-being and lifestyle choices and will enable the development of targeted interventions and public health strategies.

In recent years, Oman has witnessed a significant increase in internet usage.^15^ While internet usage is prevalent worldwide, a concerning issue arises with addiction to the internet, especially among young adults. Masters found evidence of social networking addiction among Omani students and suggested a need for intervention.^16^ However, there has been no study investigating the prevalence of internet addiction among Omani students, and no study has evaluated the potential changes in dietary patterns associated with such addiction. Although numerous studies worldwide have explored the effect of internet addiction on dietary habits, their findings cannot be generalised to Oman. It is crucial to consider the unique cultural, societal and economic factors that distinguish Omani students and conduct local research to provide tailored insights and inform effective interventions. Therefore, this study aimed to investigate the association between internet addiction and dietary habits among Omani junior college students; the authors hypothesised that internet addiction is prevalent among Omani junior college students and influences their dietary habits.

## Methods

This quantitative cross-sectional study was conducted at the College of Nursing, Sultan Qaboos University (SQU), Muscat, Oman, over a period of 4 weeks in November 2020 (during the Fall 2020 semester) among junior students between 18 and 19 years old. This age group was chosen because they are in a critical transitional phase from adolescence to early adulthood, a period marked by significant personal and academic developments that may influence internet use patterns.

The inclusion criteria included students who are aged between 18 and 19 years and enrolled in their foundation or first year at SQU. Students outside this age range were excluded. The sample size was calculated using Slovin’s formula.^17^ Based on a statistical report from SQU, the university enrols approximately 3,088 students into their foundation programme each fall semester.^18^ The formula used to compute the sample size is:


$$\text{n}=\text{N}/(1+\text{N.}{\text{e}}^{2})$$

Where N represents the population size and e represents the desired margin of error (0.05); the calculation resulted in a sample size of 354 students. To accommodate a potential 5% attrition rate, the sample size was adjusted to 370 students. A nonprobability sampling technique with a convenience sampling approach was used, selecting junior college students who were available during the data collection period. The administrators in charge of the foundation programme and first year studies at SQU were contacted and informed about the study and its objectives. With the assistance of the course coordinators, junior college students were approached.

A total of 3 instruments were used in this study. First, a socio-demographic questionnaire was used, which consisted of socio-demographic measures, including variables such as gender, age, college, year of study and grade point average (GPA).

Second, the Chen Internet Addiction Scale (CIAS), a self-reported 26-item questionnaire asks respondents to rate the degree to which each statement matches their internet use experience over the last 3 months; it assesses the primary symptoms of internet addiction, such as tolerance, compulsive use and withdrawal and evaluates the negative effects of internet addiction on social activities, interpersonal relationships, physical health and time management. The questionnaire also explores the number of hours spent online each week and the user’s level of internet experience. The scale is a 4-point Likert scale, with 1 representing ‘strongly disagree’ and 4 signifying ‘strongly agree’.^19^ The total score ranges from a 26–104, with higher scores indicating greater addiction to the internet. Chen *et al*. suggested a cut-off point of 63/64 as optimal for distinguishing cases of internet addiction from those of non-addiction, achieving a high diagnostic accuracy rate of 87.6%. Thus, students with CIAS scores exceeding 64 were classified into the internet addiction diagnosis group.^20^ The CIAS has demonstrated high reliability, with Chen *et al*.^20^ reporting a Cronbach’s alpha of 0.94 and this study confirming a high internal reliability of 0.962.

The third instrument was the Change in Dietary Habit Questionnaire, adapted from the Dietary Behaviour and Diet Quality Questionnaire developed by Kim *et al*.^21^ This questionnaire assesses recent changes in meal size, appetite, eating speed and frequency; reasons for skipping meals; and the frequency and reasons for snacking, as well as the type of snack. The first part of this questionnaire, which assesses changes in dietary habits, including meal size, appetite and eating speed while using the internet, was utilised. The original questionnaire’s Cronbach’s alpha was not reported by the authors. However, in this study, using only 3 questions from the questionnaire, the Cronbach’s alpha was 0.387.

Descriptive statistics, including frequency, percentage, mean and standard deviation, were employed to describe the participants’ demographics, the prevalence of internet addiction and the changes in eating habits. To assess the relationship between internet addiction and eating habits among adolescents, Chi-square cross-tabulation analyses were conducted. Similarly, to assess the association between internet addiction and demographics, Chi-square cross-tabulation and logistic regression were employed. Statistical analysis was performed using the Statistical Package for Social Sciences (SPSS), Version 23 (IBM Corp., Armonk, New York, USA). A *P* value of less than 0.05 was considered statistically significant.

Ethical approval was obtained from the Research and Ethics Committee of the College of Nursing at SQU. The research objectives and voluntary nature of the study were explicitly communicated to the students, who then signed a consent form and completed the questionnaires. The students were assured that their responses would remain confidential, with no names being reported or any identifying information being disclosed.

## Results

A total of 377 junior college students were recruited for this study; 55.2% were female and 44.8% were male. The average age of the students was 18.28 ± 0.45 years; the majority were 18 years old (71.9%), while the remaining (28.1%) were 19 years old. The study included students from various colleges at SQU, most of whom were from the College of Engineering (17.2%), followed by the College of Nursing (15.4%) and the College of Science (13.0%). Most of the participants were at the foundation level (70.3%). Because many of the participants were foundation-level students who had not yet finished the mandatory or elective university courses required to generate a GPA, almost half of the participants (49.9%) had no GPA [Table 1].

The diagnosis of internet addiction was made using each participant’s overall CIAS score; participants were deemed to be internet addicts if they had a CIAS score of 64 or higher. This threshold was used to estimate the prevalence rate of internet addiction among the study population; a total of 226 (59.9%) participants were identified as having internet addiction, while 151 (40.1%) participants were considered not to be addicted.

Of the junior college students who had internet addiction, the majority (62.8%) reported a change in their dietary habits, specifically a decrease in their meal size. Furthermore, 54.4% of them reported poorer appetite compared to students who maintained control over their internet use. Regarding changes in eating speed, there was no significant difference observed between internet use and eating speed among the students in either group (X^2^ = 5.687; *P* = 0.128). A statistically significant difference in both meal size (X^2^ = 30.528; *P* <0.001) and appetite changes (X^2^ = 28.731; *P* <0.001) was observed between the different levels of internet addiction among the studied junior college students [Table 2].

Chi-square cross-tabulation and logistic regression analyses were used to investigate whether there is a link between internet addiction and demographic variables, including gender, age, college affiliation and GPA. These analyses did not reveal any statistically significant results, suggesting that there is no association between these demographic factors and internet addiction in this study.

## Discussion

This study examined the prevalence of internet addiction among young junior students studying at SQU and its association with dietary habits. It is crucial to distinguish between internet addiction and necessary internet use for academic purposes. Thus, employing a validated and reliable scale is essential. The CIAS effectively identifies compulsive and detrimental internet behaviours. The authors recognise that many students depend heavily on the internet for academic research, communication and coursework completion and that this extensive, but necessary use, should not be misconstrued as addictive behaviour. According to their CIAS scores, a large percentage of junior college students at SQU (59.9%) are struggling with internet addiction. This high prevalence rate highlights the growing problem of internet addiction among young individuals, particularly in this digital age, where internet usage has become an essential component of daily life. The results are consistent with those of another study conducted in Oman among SQU students. Masters asked students to report their addiction levels and found that approximately 47.2% of them were addicted to social media, particularly YouTube.^16^ However, the current study’s results do not align with those of Far Eastern studies (China and Taiwan), where the CIAS showed an addiction prevalence ranging from 6.9% to 17.9%, which is relatively low.^22^ This discrepancy could be explained by the fact that the students included in the Far Eastern studies were above 20 years of age. Lozano-Blasco *et al*. found internet addiction to be high among young adults and reported a significant incidence among new generations.^23^

Surprisingly, more than half of the students with internet addiction (62.8%) reported a decrease in their meal size, indicating a possible link between excessive internet use and changes in dietary habits. This result is consistent with prior research that suggested that internet addiction may contribute to irregular eating patterns and meal skipping due to excessive screen time and binge gaming episodes.^10^ These poor eating habits might have a negative impact on students’ nutritional intake as well as their general health. Snacking may be associated with the high frequency of skipping dinner; more frequent snacking was reported in high-risk internet users than in low-risk internet users.^24^

Furthermore, more than half of the students with internet addiction in the current study (54.4%) reported having a worse appetite than those without addiction. This finding suggests a possible link between internet addiction and reduced appetite, which could be related to the psychological stress and isolation from real-life experiences that are typically associated with internet addiction.^25^ The results of the current study align with those of international studies showing that internet addiction among young university students is related to uncontrolled eating habits and skipping breakfast.^26^ Similarly, Hassan and Ahmed found that internet addiction in adolescents leads to loss of appetite.^10^ The current study found no significant association between internet addiction and the speed of eating among both groups. This finding suggests that, within the context of this research, internet addiction may not directly influence the speed at which students eat. However, it is important to note that other unexamined factors could affect eating speed. It is also worth considering that dietary habits are significantly influenced by local dietary culture.^27^

This study underscores the importance of counselling for junior students who struggle with internet addiction. Given the strong association between internet addiction and poor dietary habits, it is crucial to focus on comprehensive therapies that address both behavioural aspects. Addressing the underlying causes of excessive internet use is critical and should be complemented by raising awareness and implementing prevention strategies. Encouraging healthy eating habits and regular meals can also be effective in mitigating the negative effects of internet addiction on dietary patterns.^28^

This study has several limitations. Conducting the research at a single university in Oman restricts the generalisability of the results. Furthermore, the small sample size and restriction of the study population to junior students may have also impacted the findings. Moreover, the self-reporting nature of the questionnaire may have introduced response biases, such as recall bias, which relies on students’ memory of their internet use and dietary habits and may have led to inaccuracies in the reported data. Additionally, the cross-sectional nature of this study prevents the establishment of causality between internet addiction and dietary changes. Not all dietary habits identified in the literature were examined, which could inhibit a comprehensive understanding of the impact of internet addiction on dietary habits. Furthermore, because the Dietary Habits questionnaire did not follow a logical sequence in the 4-point Likert scale, it was not possible to use regression analysis to better understand the predictors of internet addiction. Finally, the CIAS does not clearly differentiate between internet use for academic and non-academic purposes, which could affect the interpretation of internet addiction levels among students.

## Conclusion

This study showed a significant prevalence of internet addiction among Omani junior college students, which was associated with negative changes in their eating habits. Nearly 60% of the students were identified as internet addicts, exhibiting dietary changes such as reduced meal size and appetite compared to their non-addicted peers. These findings emphasise the need for targeted awareness and interventions to treat internet addiction and mitigate its consequences on dietary habits.

## Figures and Tables

**Table 1:** Characteristics of the junior college students included in this study (N = 377)

Characteristic	n (%)
**Gender**	
Male	169 (44.8)
Female	208 (55.2)
**Age in years**	
18	271 (71.9)
19	106 (28.1)
**College**	
Engineering	65 (17.2)
Nursing	58 (15.4)
Science	49 (13.0)
Arts	46 (12.2)
Education	42 (11.1)
Commerce	37 (9.8)
Law	29 (7.7)
Agriculture	27 (7.2)
Medicine	24 (6.4)
**Year of study**	
Foundation	265 (70.3)
First year	93 (24.7)
Second year	19 (5.0)
**GPA**	
No GPA yet*	188 (49.9)
<2.0	20 (5.3)
2.0 – 3.0	20 (5.3)
3.1 – 3.5	108 (28.6)
3.6 – 4.0	41 (10.9

GPA = grade point average.

* First semester students are classified as ‘No GPA yet’ because they had not yet received a GPA score.

**Table 2:** Dietary habits changes among participants with and without internet addiction.

Dietary habits	Addiction Status, n (%)		X^2^	*P* value
	Internet addiction (n = 226)	No internet addiction (n = 151)		
**Changes in meal size**				
Increased	54 (23.9)	36 (23.8)	30.528	<0.001
Decreased	142 (62.8)	60 (39.7)		
No change	30 (13.3)	55 (36.5)		
**Changes in appetite**				
Better	51 (22.6)	27 (17.9)	28.731	<0.001
Worse	123 (54.4)	52 (34.4)		
No change	32 (14.2)	55 (36.4)		
Do not know	20 (8.8)	17 (11.3)		
**Changes in eating speed**				
Fast	56 (24.8)	39 (25.8)	5.687	0.128
Slow	122 (54.0)	70 (46.4)		
Average	34 (15.0)	36 (23.8)		
Irregular	14 (6.2)	6 (4.0)		
